# Risk Factors Associated With Gangrenous Cholecystitis: A Cohort Study From Eastern India

**DOI:** 10.7759/cureus.74126

**Published:** 2024-11-20

**Authors:** Karthikarajam V, Nalubolu Pushpaketu, Samit Badhai, Amaresh Mishra, Subrat Sahu, Ipsita Debata, P. K. Debata

**Affiliations:** 1 Department of General Surgery, Kalinga Institute of Medical Sciences, Bhubaneswar, IND; 2 Department of Community Medicine, Kalinga Institute of Medical Sciences, Bhubaneswar, IND

**Keywords:** acute cholecystitis, gangrene in acute cholecystitis, gangrenous cholecystitis, predicting factors, predictors of gangrenous cholecystitis

## Abstract

Background: Treating gangrenous cholecystitis (GC) can be a challenge. It necessitates urgent intervention due to its elevated mortality risk. Prompt identification of risk factors and intervention are essential for halting inflammatory cascade and preventing further complications. This study aimed to evaluate the factors for early prediction of gangrenous changes in patients with acute cholecystitis.

Methods: A prospective study was carried out among 340 diagnosed adult acute cholecystitis patients, admitted under the Department of General Surgery of a tertiary medical college between May 2022 and April 2024. Data were compiled into Excel and analyzed using the SPSS (Statistical Product and Service Solutions) software (IBM SPSS Statistics for Windows, Version 21.0. IBM Corp., Armonk, NY). Data were analyzed using descriptive statics, chi-square test, and unpaired t-test. A p-value less than 0.05 was considered statistically significant.

Results: Out of 340 acute cholecystitis patients, 27 (7.9%) progressed to GC. It was more prevalent among males (22/27, 81.5%), among patients in the 51-70 years age group (15/27, 55.5%), and among patients with comorbidities (diabetes mellitus and coronary artery disease) (23/27, 85.2%) with a significant association (p < 0.001, p = 0.010, and p < 0.001, respectively). The presence of fever (p = 0.002) and vomiting (p = 0.040) was significantly associated with gangrenous outcomes in the patients. The mean values of lab parameters like White Blood Cells (WBCs), serum bilirubin (SBIL), serum glutamic-oxaloacetic transaminase (SGOT), serum glutamic pyruvic transaminase (SGPT), and alkaline phosphatase (ALP) were significantly higher among patients with GC. Ultrasound imaging parameters showed a significantly higher asymmetrical gallbladder (GB) wall (27, 100%), intraluminal membrane (23/27, 85.2%), pericholecystic collection (23/27, 85.2%), and acalculous GB (9/27, 33.3%) among GC patients. In the case of Contrast-Enhanced Computed Tomography (CECT) for GC, the test demonstrated relatively high sensitivity (74.1%) and specificity (85.6%). All patients with GC underwent emergency cholecystectomy.

Conclusion: The study highlighted various demographic, clinical, and imaging factors linked to a high risk of developing GC, including older age, male gender, elevated laboratory markers (such as WBC count, SBIL, SGOT, SGPT, and ALP), and imaging features like GB calculi, increased GB wall thickness, wall symmetry, intraluminal membrane, and pericholecystic collection. Employing a comprehensive approach that incorporates demographic, clinical, and imaging data is essential for predicting and guiding treatment decisions effectively.

## Introduction

Gangrenous cholecystitis (GC) is a severe complication of acute cholecystitis [1]. Studies have found that GC occurs unexpectedly in a range of 2% to 29.6% of cases of acute cholecystitis [2,3]. The incidence of GC is 5.6% and 31.2% in acute calculous cholecystitis (ACC) and acute acalculous cholecystitis (AAC) respectively [4,5].

Prolonged cystic duct obstruction leads to epithelial injury, causing progressive vascular insufficiency. This ischemia leads to necrosis and potentially perforation of the gallbladder (GB) wall, resulting in GC. This epithelial injury triggers the release of phospholipases, enzymes that degrade nearby cell membranes, leading to a pronounced inflammatory response. The combined effect of increased tension in the GB wall and the heightened inflammatory reaction ultimately leads to either localized or widespread ischemia of the GB wall [1,3].

Sonography and Computed Tomography (CT) imaging techniques effectively identify gangrenous changes associated with acute cholecystitis. Sonography is commonly employed as the initial imaging modality due to its high sensitivity, which stands at 97%, albeit with a specificity of 76%. On the other hand, CT imaging offers high specificity and is particularly valuable in cases where the findings are inconclusive or ambiguous [4,6,7]. Patients exhibiting either focal transmural necrosis of the GB or diffuse necrosis in imaging are diagnosed with GC. Other diagnostic indicators include the presence of air within the GB wall, intraluminal membranes, and pronounced irregularity or septation, along with the development of pericholecystic collections within the GB wall [6-8].

GC necessitates urgent intervention due to its elevated mortality risk. Patients with GC, especially those with accompanying diabetes mellitus (DM), exhibit elevated levels of liver enzymes (alanine aminotransferase, aspartate aminotransferase, alkaline phosphatase (ALP)), as well as total bilirubin. Additional indicators of mortality risk include the detection of pericholecystic fluid during ultrasonography (USG) and the necessity to convert from laparoscopic to open surgery due to technical challenges. Delayed hospital admission and low white blood cell (WBC) counts (<4,000 cell/mm3) are identified as independent risk factors contributing to mortality in cases of GC. It is anticipated that the rate of conversion to open surgery is higher in cases of GC compared to simple acute cholecystitis or symptomatic cholelithiasis. Nevertheless, when laparoscopic cholecystectomy is completed successfully, it is linked to markedly improved outcomes and a shorter duration of hospitalization [1]. A conversion rate higher than that for simple acute cholecystitis or symptomatic cholelithiasis is to be expected. However, when successful, laparoscopic cholecystectomy is associated with a significantly better outcome and a shorter hospital stay [2].

Factors such as GB sonographic wall thickness measuring 5.1-6 mm or greater than 6 mm, male gender, presence of DM, leukocytosis exceeding 15,000 cells/ml, and age 40 years or older have been identified as predisposing factors for gangrene complicated ACC, showing a significant statistical variance. Additionally, elevated levels of ALT, AST, and ALP could also assist in making decisions regarding early surgical intervention [8]. There were no statistically significant differences in the overall therapeutic outcomes between AAC and ACC, regardless of the treatment method used. However, the recurrence rate following nonsurgical treatment was notably lower in the AAC group compared to the ACC group [9]. While risk factors for GC such as age, gender, and select lab parameters are established, this study provides a regional perspective from Eastern India, highlighting variations in prevalence and clinical presentation specific to this population as well as supporting the previous studies. Additionally, it identifies imaging findings (e.g., asymmetrical GB wall and intraluminal membrane presence) as predictive markers, contributing to a more comprehensive diagnostic profile for early GC detection. This study aims to evaluate the factors for early prediction of gangrenous changes in patients with acute cholecystitis.

## Materials and methods

Study design, population, and setting

A prospective cohort study was carried out by the Department of General Surgery of a tertiary medical college and hospital, among patients diagnosed with acute cholecystitis who were admitted to the hospital between May 2022 and April 2024.

Inclusion criteria

Patients, aged 18 years and above, admitted with acute cholecystitis, diagnosed through a comprehensive evaluation encompassing clinical, laboratory, and imaging procedures, were included in the study.

Exclusion criteria

Patients with concurrent conditions such as common bile duct (CBD) stone, primary sclerosing cholangitis, acute pancreatitis, mesenteric vascular thrombosis, hypercoagulable state, acute cholecystitis with multiple organ dysfunction syndrome (MODS)/septic shock, and major burns were excluded. Additionally, patients with suspected GB carcinoma or other malignancies were also excluded from the study.

Sample size and sampling technique

The study participants were selected by a universal sampling technique. A total of 340 patients were enrolled during the study duration. Consecutive enrollment of eligible patients scheduled for surgery was adopted to minimize selection bias. This approach ensured the inclusion of all eligible individuals meeting the predetermined criteria, enhancing the generalizability of study findings. The study aimed to capture a diverse range of cases, thereby contributing to a comprehensive understanding of factors influencing the early detection of GC.

Data collection

Data collection involved a multi-step process. Initially, patients meeting the inclusion criteria underwent an ultrasound examination of the abdomen and pelvis to assess the status of the GB. Radiology reports were subsequently compared with intraoperative findings and histopathology reports of resected specimens. Data were meticulously recorded using a pretested case record Performa specifically designed for the study. Following discharge, patients were advised to undergo follow-up evaluation after a week post-surgery at the Department of General Surgery.

Elimination of bias

To mitigate bias, especially selection bias, all eligible patients meeting the inclusion and exclusion criteria were included in the study. This comprehensive approach aimed to ensure the representation of diverse patient profiles, enhancing the validity and generalizability of study findings. Furthermore, measures were implemented to minimize information bias by meticulously measuring and cross-checking key study variables during data collection. By adhering to rigorous data collection protocols, the study aimed to enhance the reliability and accuracy of its findings, thereby strengthening the overall robustness of the research outcomes.

Data analysis

Data was compiled into Excel and analyzed using the SPSS (Statistical Product and Service Solutions) software (IBM SPSS Statistics for Windows, Version 21.0. IBM Corp., Armonk, NY). Descriptive data were represented in mean ± SD, frequencies, and percentages. The chi-square test was used to test association. To compare mean values between groups, the Unpaired t-test was employed. A p-value less than 0.05 was considered statistically significant.

Ethical considerations

Institutional Ethical Clearance (Ref no.: KIIT/KIMS/IEC/1010/2022) was obtained before commencing the study and the study was conducted as per the Declaration of Helsinki. Informed consent was obtained from all participants, and confidentiality was strictly maintained throughout the study. Patients were provided with a comprehensive overview of the study in a language they understood, and their identities were safeguarded at all times. Furthermore, the study was conducted in a manner that did not hinder patients' routine activities. Data obtained during the study were recorded conscientiously and securely to ensure the integrity and confidentiality of participants' information.

## Results

**Figure 1:**
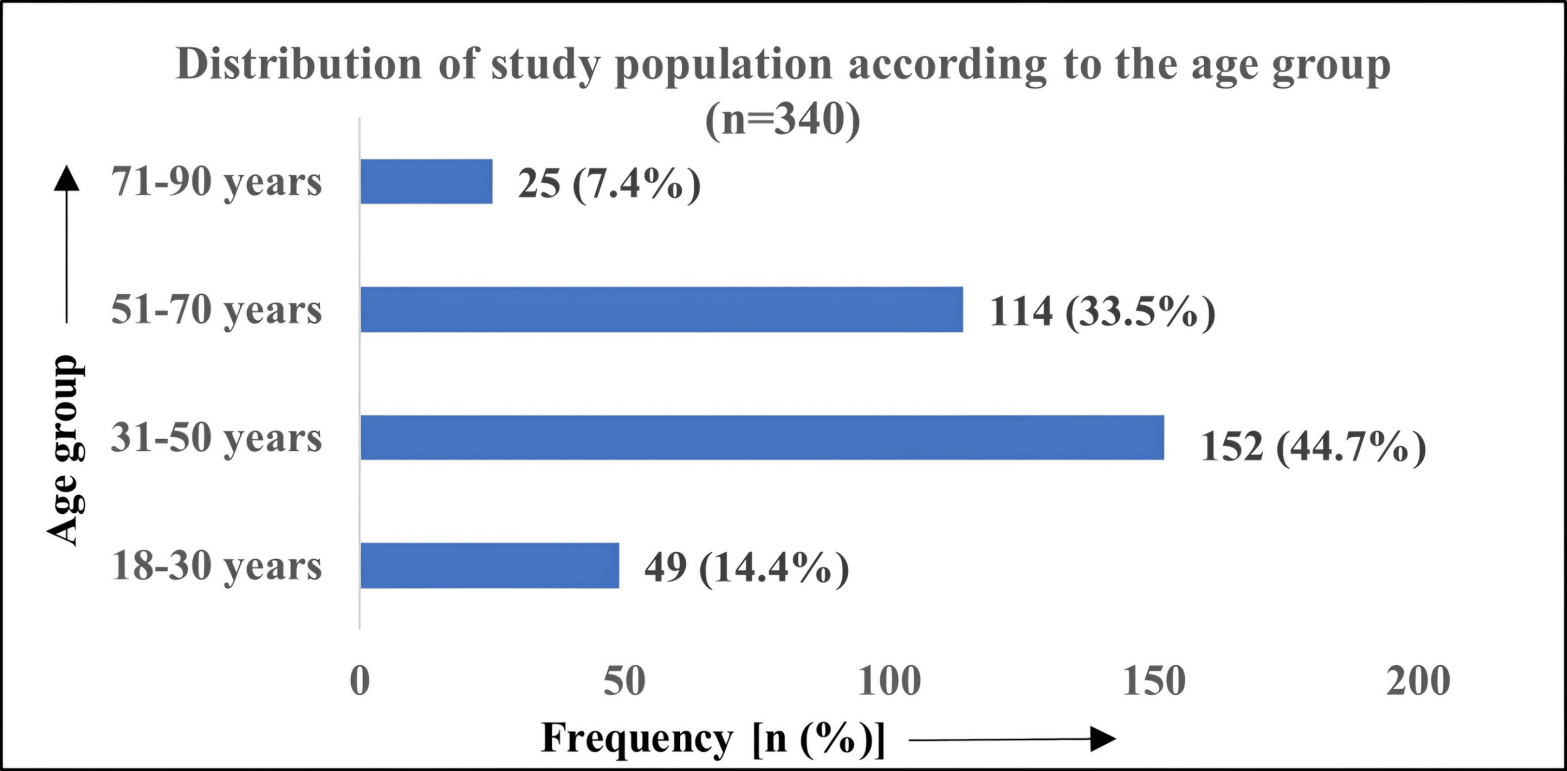
Distribution of study participants according to age group (n=340)

A total of 340 patients with acute cholecystitis were included in the study. There were 200 (58.8%) females and 140 (41.2%) males. Around 152 (44.7%) were between 31 and 50 years of age, followed by 114 (33.5%) belonging to the 51-70 age group. Figure 1 shows the distribution of study participants according to the age group.

In the study, out of 340 acute cholecystitis patients, 27 (7.9%) progressed to GC while 313 (92.1%) did not experience gangrenous complications. GC was more commonly seen among males (22/27, 81.5%) than females (5/27, 18.5%). The incidence of gangrenous outcome among acute cholecystitis was higher for patients in the 51-70 years age group (15/27, 55.5%), and among patients with comorbidities (23/27, 85.2%). Table 1 shows the distribution of GC among the study participants according to gender, age group, and comorbidity status (DM and coronary artery disease) and their association.

**Table 1:** Distribution of study participants according to gangrenous outcome and their association p < 0.05 considered statistically significant

Variable	Gangrenous cholecystitis (n=340)		χ^2^ (p-value)
	Yes (n=27), N (%)	No (n=313), N (%)	
Gender			
Male	22 (81.5)	118 (37.7)	19.7 (p < 0.001)
Female	05 (18.5)	195 (62.3)	
Age group (in years)			
18-30	01 (3.7)	48 (15.3)	11.1 (p = 0.010)
31-50	07 (25.9)	145 (46.3)	
51-70	15 (55.6)	99 (31.7)	
71-90	04 (14.8)	21 (6.7)	
Comorbidity status			
Present	23 (85.2)	97 (31)	31.9 (p < 0.001)
Absent	04 (14.8)	216 (69)	

The most common symptom among the admitted patients was pain abdomen (assessed by the visual pain scale) (300/340, 88.2%), followed by fever (128/340, 37.6%). The presence of fever (p = 0.002) and vomiting (p = 0.040) was significantly associated with gangrenous outcomes in the patients, as depicted in Table 2.

**Table 2:** Association of symptoms with gangrenous cholecystitis p < 0.05 considered statistically significant

Variable	Gangrenous cholecystitis (n=340)		χ^2^ (p-value)
	Yes (n=27), N (%)	No (n=313), N (%)	
Pain			
Yes	26 (96.3)	274 (87.5)	1.8 (p = 0.170)
No	01 (3.7)	39 (12.5)	
Fever			
Yes	19 (70.4)	110 (35.1)	13.1 (p = 0.002)
No	08 (29.6)	203 (64.9)	
Vomiting			
Yes	14 (51.8)	102 (32.6)	4.1 (p = 0.040)
No	13 (48.2)	211 (67.4)	

All 340 enrolled patients underwent USG, representing 100% of the study cohort. Around 65 (19.1%) patients received contrast-enhanced CT (CECT) to obtain detailed anatomical information and assess vascular involvement in suspected cases of GC and around 58 (17.1%) patients underwent magnetic resonance cholangiopancreatography (MRCP) to visualize the biliary tract and pancreatic ducts, aiding in the diagnosis and characterization of biliary pathologies, including cholecystitis. In the case of CECT for GC, the test demonstrated relatively high sensitivity (74.1%) and specificity (85.6%), suggesting its utility in identifying individuals with the condition and excluding those without it. In the case of MRCP for detecting GC, it demonstrated relatively low sensitivity (29.6%) but moderate specificity (84.0%), as depicted in Table 3.

**Table 3:** Association between gangrenous cholecystitis and type of investigation CECT - contrast-enhanced computed tomography, MRCP - magnetic resonance cholangiopancreatography p < 0.05 considered statistically significant

Type of investigation	Gangrenous cholecystitis (n=340)		χ^2^ (p-value)
	Yes (n=27), N (%)	No (n=313), N (%)	
CECT			
Yes	20 (74.1)	45 (14.4)	57.3 (p < 0.001)
No	07 (25.9)	268 (85.6)	
MRCP			
Yes	08 (29.6)	50 (16)	3.3 (p = 0.070)
No	19 (70.4)	263 (84)	

In the study, the average hospital stay for acute cholecystitis was 4.91 days whereas in GC, the average stay significantly increased to 12.44 days, suggesting the increased morbidity rate in patients with GC. The mean values of lab parameters like WBCs, serum bilirubin (SBIL), serum glutamic-oxaloacetic transaminase (SGOT), serum glutamic pyruvic transaminase (SGPT), and ALP were significantly higher among patients with GC than those with only acute cholecystitis (AC), as depicted in Table 4. These findings underscored these laboratory parameters' potential diagnostic and prognostic value in identifying and predicting GC.

**Table 4:** Comparison of hospital stay and lab parameters between two groups by independent t-test GC - Gangrenous cholecystitis, AC - Acute cholecystitis p < 0.05 considered statistically significant

Variable	Groups	Mean (± SD)	t-test (p-value)
Hospital Stay	GC	12.44 (± 6.31)	16.47 (<0.001)
	AC	4.91 (± 1.52)	
White blood cell (WBC)	GC	20,920.59 (± 4418.72)	10.14 (<0.001)
	AC	14,286.10 (± 3146.22)	
Serum Bilirubin	GC	2.82 (± 0.70)	5.61 (< 0.001)
	AC	1.98 (± 0.75)	
Serum glutamic-oxaloacetic transaminase (SGOT)	GC	85.63 (± 16.42)	4.23 (< 0.001)
	AC	71.28 (± 16.93)	
Serum glutamic-pyruvic transaminase (SGPT)	GC	74.29 (± 20.46)	6.76 (< 0.001)
	AC	55.44 (± 13.20)	
Alkaline phosphatase (ALP)	GC	175.41 (± 28.47)	5.77 (< 0.001)
	AC	140.84 (± 29.97)	

Based on imaging parameters as shown in Table 5, it was reported that all 27 patients with GC had asymmetrical gall bladder (GB) walls highlighting this finding as one of the sensitive parameters to suspect gangrenous changes. The intraluminal membrane was present in 23 out of 27 patients of GC, whereas it was not seen in any case of acute cholecystitis, suggesting this feature on imaging to be more specific for gangrenous outcome. The pericholecystic collection was seen in 23 out of 27 cases of GC and 112 out of 313 patients of acute cholecystitis, which was statistically significant. From this, we infer that pericholecystic collection is a predominant feature seen in acute cholecystitis, but it can be present in GC patients too. Of the 27 patients with GC, nine (33.3%) had GB calculus. In contrast, among the 313 patients without GC, 287 patients (91.7%). This association was found to be significant which implies that GC occurrence is higher in patients with acalculous cholecystitis.

**Table 5:** Imaging parameters and their association with gangrenous cholecystitis as an outcome p < 0.05 considered statistically significant

Imaging parameters		Gangrenous cholecystitis		χ^2^ (p-value)
		Yes (n=27), N (%)	No (n=313), N (%)	
GB wall symmetry	Asymmetrical	27 (100)	0 (0)	301.34 (< 0.001)
	Diffuse thickness	0 (0)	26 (8.3)	
	Symmetrical	0 (0)	287 (91.7)	
Intraluminal membrane	Absent	04 (14.8)	313 (100)	270.53 (< .001)
	Present	23 (85.2)	0 (0)	
Pericholecystic collection	Absent	04 (14.8)	201 (64.2)	25.34 (< .001)
	Present	23 (85.2)	112 (35.8)	
GB calculus	No	18 (66.7)	26 (8.3)	75.14 (< .001)
	Yes	9 (33.3)	287 (91.7)	

On imaging, the mean GB wall thickness was 4.75 (± 0.83) mm in patients with only acute cholecystitis, and 6.5 (± 1.30) mm in patients who progressed to GC, and this association was found to be statistically significant. These findings highlighted that any acute cholecystitis patient with a GB wall thickness of more than 6.5 mm should be evaluated for GC.

**Figure 2:**
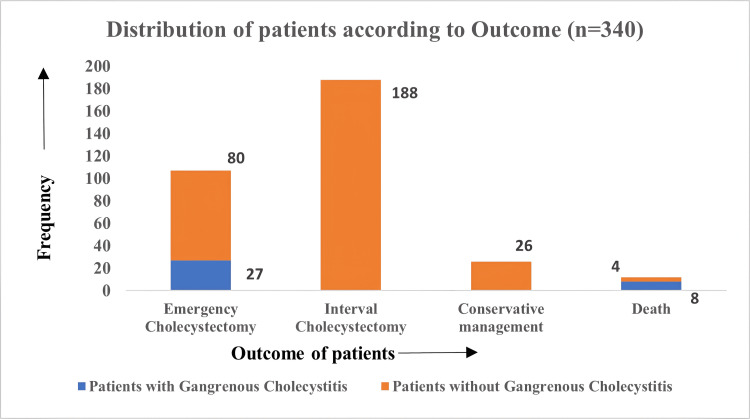
Frequency distribution of study participants according to the outcome

On assessing the outcome of the enrolled patients, a total of 107 (31.5%) patients underwent emergency cholecystectomy (EC), which included all the patients with GC (27, 100%). Around 188 (55.3%) patients underwent interval cholecystectomy (IC), and 25 (7.6%) patients were managed conservatively (including measures such as antibiotic therapy, fluid resuscitation, and supportive care aimed at stabilizing the patient's condition without surgical intervention). The outcome is depicted in Figure 2.

## Discussion

General surgeons frequently treat routine AC appropriately, but treating GC safely is more difficult and carries a much higher risk of morbidity. The study included 340 acute cholecystitis patients, with 58.8% females and 44.7% aged between 31 and 50 years of age. Around 7.9% of the study population progressed to develop GC. GC was more prevalent among males (22/27, 81.5%) than females (5/27, 18.5%). The incidence of gangrenous outcome among acute cholecystitis was higher for patients in the 51-70 years age group (15/27, 55.5%), and among patients with comorbidities (23/27, 85.2%). This finding corroborates the findings of Gomes et al. who also reported a higher prevalence (29.3%) of GC among the male gender and with age more than 50 years old and among patients with DM (p = 0.006) [10]. Thus, males are more predisposed to severe forms of cholecystitis, including gangrenous and complicated cases. The underlying mechanisms may include differences in gallstone composition, hormonal influences, and genetic predisposition on which further research is required. Additionally, lifestyle factors such as dietary habits and alcohol consumption may also contribute to the observed sex differences in severe cholecystitis prevalence. The study also highlighted individuals aged 51-70 years as being at the highest risk for GC. This age group likely represents a population with a higher prevalence of comorbidities, decreased physiological reserve, and increased susceptibility to complications, all of which contribute to the heightened risk of developing severe cholecystitis. Several other studies have also reported male gender and higher age group (45-50 years) as notable risk factors for GC [11,12].

In the study, the average hospital stay for acute cholecystitis was 4.91 days whereas in GC, the average stay significantly increased to 12.44 days, suggesting the increased morbidity rate in patients with GC. The mean values of lab parameters like WBCs, SBIL, SGOT, SGPT, and ALP were significantly higher among patients with GC than those with only acute cholecystitis (AC). The higher WBC count indicates more severe inflammation and a higher risk of complications like gangrene and perforation. The higher SBIL levels among patients with GC might be due to biliary obstruction from GB inflammation or necrosis. The elevated SGOT, SGPT, and ALP levels in GC patients may reflect liver involvement due to biliary obstruction or systemic inflammation related to severe disease. Similar findings were reported by Yacoub et al. who marked gall bladder necrosis in acute cholecystitis patients with leukocytosis (> 13,000/mm3) [13]. In their retrospective analysis, another study by Fagan et al. also found that GC was linked to African American race, age ≥51 years, WBC count ≥15,000, diabetes, pericholecystic fluid, aspartate aminotransferase, alanine aminotransferase, ALP, and lipase. Multivariate analysis highlighted diabetes and WBC count as independent risk factors for GC [14]. This study emphasized identifying these risk factors in predicting GC and advocating urgent surgical intervention in high-risk patients. Another study by Aydin et al., in their retrospective analysis, revealed an independent association of male sex, DM, and WBC count with the development of acute GC. Specifically, male patients with diabetes and a WBC count greater than 14,900/mm³ were found to be at increased risk for GC, highlighting the importance of early recognition and prompt surgical management in these high-risk individuals [15]. The laboratory findings can aid clinicians in risk stratification, guiding treatment decisions, and predicting patient outcomes in cases of acute cholecystitis. By integrating laboratory parameters with clinical findings and imaging studies, clinicians can develop a comprehensive approach to the diagnosis and management of GC, ultimately improving patient care and outcomes. Further research is warranted to validate these findings and elucidate the optimal diagnostic algorithms for early detection and management of GC.

In this study, asymmetrical GB wall symmetry, the presence of intraluminal membrane and pericholecystic collection, and acalculous cholecystitis were significantly associated with GC. Thickened GB walls (≥ 6.5 mm in this study), indicative of inflammation and edema, were linked considerably to GC, emphasizing their role as markers of disease severity. The study underscores the intricate relationship between gallstone disease, GB wall thickening, and the development of severe cholecystitis. Overall, these findings challenge established assumptions about imaging features in GC. Clinicians should interpret imaging results in the context of clinical symptoms and consider alternative diagnostic approaches when suspecting GC despite inconclusive imaging findings. A study by Wu et al. also reported in their risk assessment models that pericholecystic fluid and GB wall thickening (≥ 4 mm) were significantly associated with GC [16].

In the study, the use of different imaging modalities, namely USG, CECT, and MRCP were assessed to determine their diagnostic utility in identifying GC cases. USG emerged as the most commonly utilized imaging modality in the study cohort, with universal performance across all patients. USG is often considered the initial imaging modality of choice for evaluating GB pathology due to its widespread availability, non-invasiveness, and ability to provide real-time imaging of the GB and surrounding structures. However, its ability to differentiate between uncomplicated and GC may be limited, particularly in cases with equivocal findings [17].

In the study, CECT (high sensitivity and specificity) and MRCP (low sensitivity and moderate specificity) had a significant association with the diagnosis of GC. In cases of suspected GC, CECT can provide valuable information regarding the presence of GB wall necrosis, pericholecystic inflammation, and complications such as perforation or abscess formation. While MRCP is valuable for evaluating biliary anatomy and detecting CBD stones or strictures, its role in the diagnosis of acute cholecystitis, including gangrenous disease, is less well-established. MRCP may be reserved for cases where there is clinical suspicion of biliary obstruction or concurrent pancreatic pathology but is not routinely used as a primary imaging modality for evaluating GC [18].

A retrospective analysis by Bennet also highlighted the significant association between specific CT features (intraluminal membranes, gas in lumen, irregular wall, and pericholecystic abscess) and the diagnosis of GC, with a diagnostic accuracy of 86.7%, emphasizing the role of CT imaging in identifying patients at risk of developing severe cholecystitis complications [19]. Another study by Chang also reported high specificity (93.9%) and accuracy (90.9%) of CT in diagnosing GC, underscoring the value of CT imaging as a reliable tool for early detection and risk stratification in cholecystitis patients [4]. Further research and standardization of imaging protocols are warranted to optimize the diagnostic accuracy and clinical utility of imaging modalities in the management of GC.

The study highlighted various demographic, clinical, and imaging factors linked to a high risk of developing gangrenous changes, including older age, male gender, elevated laboratory markers (such as WBC count, SBIL, SGOT, SGPT, and ALP), and imaging features like GB calculi, increased GB wall thickness, wall symmetry, intraluminal membrane, and pericholecystic collection. Clinicians need to be vigilant in assessing patients with risk factors for GC and should consider early surgical consultation for those at high risk. Prompt surgical intervention, such as cholecystectomy, is often necessary to remove the infection source and prevent complications like perforation or sepsis. Close monitoring of clinical and imaging findings is crucial to detect disease progression in severe cholecystitis and guide treatment escalation as needed.

Additionally, the study stresses the importance of ongoing research into supplementary clinical and imaging indicators that could improve the prediction and diagnosis of GC. The study highlights the limited research on symptom duration as a predictor for GC. Moreover, exploring new biomarkers, such as inflammatory markers or advanced imaging technologies, may offer additional prognostic information and facilitate early detection of the disease to enhance patient care and reduce the morbidity and mortality associated with GC.

Limitation

The study did not explore the association between clinical signs and symptoms with the outcome of acute cholecystitis in detail. Exploring this association could have shed more clarity on other possible predictors for gangrenous outcomes in acute cholecystitis patients. Further upscaled research could be taken up and predictive modeling with multivariate analysis could be done to identify the independent effect of each predictor on the risk of gangrene. This would provide better results by isolating specific risk factors more effectively.

## Conclusions

A high index of suspicion is necessary for the early diagnosis and treatment of GC in patients with acute cholecystitis. This study, conducted within the Eastern Indian population, underscores the prevalence of specific risk factors - male gender, advanced age, and comorbidities such as DM and cardiovascular diseases - that may reflect regional healthcare access patterns and lifestyle factors. Laboratory parameters, including leukocytosis and elevated levels of SGOT, SGPT, and ALP, also serve as significant predictors of GC in this population. Diagnostic imaging, particularly USG and CECT played a critical role in assessing cholecystitis severity, with USG parameters like thickened GB wall, intraluminal membrane presence, pericholecystic collection, and acalculous cholecystitis proving to be valuable indicators. CECT, with its high sensitivity and specificity, provides essential information on complications such as GB wall necrosis, pericholecystic inflammation, and possible perforation or abscess formation.

This study highlights the importance of early surgical intervention in GC cases, evidenced by the higher incidence of emergency cholecystectomy in these patients. Prompt surgical consultation and intervention are vital for halting the inflammatory cascade and preventing further complications. Integrating demographic, clinical, laboratory, and imaging data allows for a comprehensive approach to guide treatment decisions effectively in Eastern India, where timely intervention can significantly impact patient outcomes.
